# Editorial: Computational tools in inferring cancer tissue-of-origin and molecular classification towards personalized cancer therapy, volume III

**DOI:** 10.3389/fgene.2022.1078871

**Published:** 2022-11-18

**Authors:** Yinglu Guo, Xiaoyan Liu, Yi Liu, Hantao Zhang, Junlin Liu, Chaofan Shan, Xun Gong, Min Tang

**Affiliations:** ^1^ School of Life Sciences, Jiangsu University, Zhenjiang, Jiangsu, China; ^2^ Institute of Animal Science, Jiangsu Academy of Agricultural Sciences, Nanjing, China; ^3^ Affiliated Hospital of Jiangsu University, Zhenjiang, China

**Keywords:** cancer, molecular classification, machine learning, biomarkers, prognosis

As one of the severe diseases threatening the health of human being, the cancer has an increasing incidence and mortality. To a certain extent, cancer mortality can be reduced if cases are classified and treated correctly. During the past decades, computational tools have showed significant advantages in classification, diagnosis, and prognosis of cancer types. Although there have been several computational methods developed in this area, the accuracy is yet to be improved to sure a clinical application. Taken together, it is required that more studies that can develop effective signatures and novel computational tools to build the personalized cancer therapy. The Research Topic here introduces not only many kinds of methods or tools to infer cancer tissue-of-origin and molecular classification, but also covers translational studies for cancer treatment in hospitals. The 26 published articles consist of 25 original research papers and a case report, which illustrates the use of computational tools in discovering molecular biomarkers and establishing more accurate prognostic models in various cancer types, including but not limited to breast cancer (BC), colorectal cancer (CRC), gastric cancer (GC), hepatocellular carcinoma (HCC), stomach cancer (SC), lung adenocarcinoma (LUAD), esophageal cancer (EC), glioma and pancreatic cancer (PC).

Of which, eight studies focused on the development of computational approaches. Huang et al. established multi-parametric MRI-based radiomics models to differentiate molecular subtype and androgen receptor (AR) expression in BC. They used the leave-one-out cross-validation (LOOCV) method to construct machine learning models. Then, they applied six supervised classification algorithms and applied the receiver operating characteristic (ROC) curve to demonstrate the model performance. A similar study in CRC was completed by Hu et al. In their project, they built radiomics models based on different phase CT images for predicting Kirsten rat sarcoma virus (KRAS) mutation in patients with CRC. Moreover, Lu et al. proposed a novel framework that uses Extreme Gradient Boosting (XG Boost) to trace the primary site of cancer of unknown primary site (CUP) based on microarray-based gene expression data. In addition, Niu et al. explored a deep learning model to predict tumor mutation burden (TMB) based on histopathological images of LUAD. And Su et al. served a novel computational framework for predicting the survival of cancer patients with PD-1/PD-L1 checkpoint blockade therapy. Besides, nomogram survival prediction models were developed to predict the prognosis of HCC after invasive treatment (Zhang S.W et al.). And an algorithm developed to combine polymorphisms in cytochrome P450 genes and clinicopathological signatures to identify a subpopulation of BC patients (Pang et al.). Besides, Miao et al. developed a novel computational method to predict tissue-of-origin of cancer of unknown primary patients by explicitly integrating expression quantitative trait loci (eQTL) into an XGBoost classification model. Notably, twelve studies aimed to reveal the novel prognosis-related signatures in different cancers. For instance, Lai et al. considered the important role of lncRNAs in epigenetic regulation and protein-coding gene regulation. Hence a prognostic model with good predictive performance based on 10 ferroptosis-related lncRNAs was constructed. Similarly, Xiao et al. indicated that immune ferroptosis-related genes might be potential predictors of STAD’s response to ICI immunotherapy biomarkers. Based on gene expression profiles, a highly survival-associated five-gene risk score model was established by Chen et al. to predict the prognosis of multiple myeloma patients (Chen et al.). And Huang et al. explored a potential association between metabolism and cell renal cell carcinoma (ccRCC). They established model could serve as an independent prognostic biomarker, provide potential therapeutic targets for the clinical treatment of RCC. Besides, Lysine (K)-specific demethylase 6B (KDM6B) is an epigenetic enzyme involved in the coordinated control between cellular intrinsic regulators. Ding et al.’s study offered a relatively comprehensive understanding of KDM6B’s role in cancer development. Huang et al. suggested that coiled-coil domain containing 134 (CCDC134) can serve as a biomarker of poor prognosis and a potential immunotherapy target in BC. Likewise, cell cycle checkpoints related genes (CCCRGs) signature have potential utility in predicting patient outcomes, and response to immunotherapies and chemotherapies for LUAD patients (Yang et al.). And the metabolic pathway phenotypes may predict overall survival excellently for HCC patients (Ye et al.). Apart from that, TIMM 8A could be clarified as a biomarker for poor prognosis of BC and a potential target of immunotherapy (Zhang Y et al.). And the mitochondrial-associated protein leucine-rich pentatricopeptide repeat-containing (LRPPRC) may act as an oncogene *via* maintaining mitochondrial homeostasis and could be used as a predictive marker for patient prognosis in PC (Wang et al.). Especially, 11-gene panel is suitable for molecular classification in grade 3 endometrial endometrioid carcinoma and for guiding prognosis (Li L et al.). Then it is showed that KRT19P3 could be used as a marker to differentiate BC from para cancer tissue (Fan et al.).

Furthermore, two studies have screened and identified genes associated with cancer diagnosis and treatment. By weighted gene co-expression network analysis, eight hub genes were finally identified to be closely correlated with LUAD recurrence (Shen et al.). Several immune-related genes and immune cell subtypes related to the neoadjuvant chemotherapy response were identified, and further verified the importance of immunotherapy combined with chemotherapy (Zhou et al.). In addition, three studies have developed or benefited from sequencing technologies. Zhuang et al. proposed a novel single-cell RNA sequencing (scRNA-seq) data analysis method based on gene function enrichment analysis to divide genes into different gene functional modules and to extract the characteristics of the cells from these functional feature matrices. And a novel method was developed to detect microsatellite instability (MSI) based on next-generation sequencing (NGS) data (Li S et al.). And Lin et al. used whole-exome sequencing (WES) to explore the differences in the evolution map and heterogeneity in different regions and detect tumor-specific mutations in patients to help improve the prognosis of EC patients. Finally, one case report discussed the tumor mutation burden as an indicator to predict efficacy of immune checkpoint inhibitors. In this report, they used one advanced lung squamous cell carcinoma case as a discussion of the significance of TMB (Wu et al.).

In conclusion, the research articles and case report in this Research Topic had guiding significance for inferring cancer tissue-of-origin and molecular classification. The potential applications of individualized cancer treatment have been widely described in these articles. At the same time, we are also looking forward to more new methods in cancer molecular classification, diagnosis, prognosis, and treatment.

